# Analysis of Vipadenant and Its In Vitro and In Vivo Metabolites via Liquid Chromatography-Quadrupole-Time-of-Flight Mass Spectrometry

**DOI:** 10.3390/pharmaceutics10040260

**Published:** 2018-12-03

**Authors:** Seok-Ho Shin, Min-Ho Park, Jin-Ju Byeon, Byeong ill Lee, Yuri Park, Nahye Kim, Jangmi Choi, Young G. Shin

**Affiliations:** College of Pharmacy and Institute of Drug Research and Development, Chungnam National University, Daejeon 34134, Korea; seokho.shin.cnu@gmail.com (S.-H.S.); minho.park.cnu@gmail.com (M.-H.P.); jinju.byeon.cnu@gmail.com (J.-J.B.); byungill.lee.cnu@gmail.com (B.i.L.); yuri.park.cnu@gmail.com (Y.P.); nahye.kim.cnu@gmail.com (N.K.); jangmi.choi.cnu@gmail.com (J.C.)

**Keywords:** vipadenant, A2a receptor antagonist, immune checkpoint, LC-QTOF-MS, metabolite identification, pharmacokinetics

## Abstract

A simple and sensitive liquid chromatography–quadrupole-time-of-flight–mass spectrometric (LC-QTOF-MS) assay has been developed for the evaluation of drug metabolism and pharmacokinetics (PK) properties of vipadenant in rat, a selective A2a receptor antagonist as one of the novel immune checkpoint inhibitors. A simple protein precipitation method using acetonitrile was used for the sample preparation and the pre-treated samples were separated by a reverse-phase C18 column. The calibration curve was evaluated in the range of 3.02 ~ 2200 ng/mL and the quadratic regression (weighted 1/concentration) was used for the best fit of the curve with a correlation coefficient ≥0.997. The in vivo PK studies in rats showed that vipadenant bioavailability was 30.4 ± 8.9% with a low to moderate drug clearance. In addition, in vitro/in vivo metabolite profiles in rat were also explored. Five different metabolites were observed in our experimental conditions and the major metabolites were different between in vitro and in vivo conditions. As far as we know, there has been no report on the development of quantitative methods for its PK samples nor the identification of its metabolites since vipadenant was developed. Therefore, this paper would be very useful to better understand the pharmacokinetic and drug metabolism properties of vipadenant in rat as well as other species.

## 1. Introduction

Recently, many studies have emphasized the importance of the immune checkpoint pathways using PD-1/PD-L1 and CTLA-4 inhibitors as novel anticancer strategies [1,2,3,4,5,6,7]. In addition, significant interests were also paid to the adenosine A2a receptor because it plays a critical role for adenosine which presents in the tumor microenvironment to activate the negative immunity feedback loop [8,9,10].

**Figure 1:**
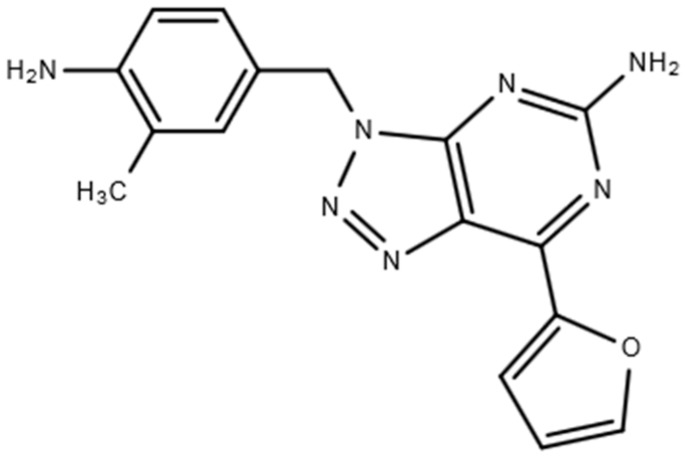
Structure of vipadenant.

Vipadenant (Figure 1) is a selective inhibitor of the A2a receptor, originally developed by Vernalis as an oral drug for the treatment of Parkinson’s disease (PD). After clinical Phase I and Phase II trials in PD patients, vipadenant was licensed out to RedoxTherapies because of its potent efficacy as a therapeutic agent in combination with immunotherapy agents [11]. There have been some studies reported regarding the efficacy [12,13,14,15,16] or pharmacodynamics of vipadenant in the pre-clinical models [16,17] or human trials assessing the A2a receptor occupancy [18]. However, to our best knowledge, no studies in terms of the pharmacokinetics (PK) or the metabolic profiles of vipadenant. We therefore developed a simple and sensitive LC-QTOF-MS assay to quantify the concentration of vipadenant from in vivo rat plasma samples for the characterization of its PK properties in rat. The identification of metabolites from in vitro/vivo samples for vipadenant was also conducted using the high resolution TOF mass spectrometer. 

The qualification of the assay was performed with respect to the precision, accuracy, dilution integrity, stability, recovery and species-dependent matrix effect and so forth. The assay was successfully applied to determine the PK parameters of vipadenant in rats after intravenous or oral administration. Also, the metabolite identification (MetID) studies were conducted both in vitro (using rat liver microsomes) and in vivo (using rat PK samples) and the results of the metabolites revealed in each study were compared.

## 2. Materials and Methods

### 2.1. Reagents & Chemicals

Vipadenant was acquired from Medchem Express (Monmouth Junction, NJ, USA). Tween 20, dimethyl sulfoxide (DMSO), formic acid, methanol (MeOH), HPLC grade acetonitrile (ACN) and HPLC grade distilled water (DW) were all obtained from Daejung Chemical (Siheung, Gyeonggi-Do, Korea). 

Rat liver microsome (Sprague-Dawley, male) was purchased from Corning (Tewksbury, MA, USA). Glutathione (GSH), uridine 5′-diphosphoglucuronic acid triammonium salt (UDPGA), nicotinamide adenine dinucleotide phosphate reduced (NADPH) and all other chemical/reagents used for MetID and metabolite profiling were purchased from Merck & Sigma-Aldrich (Yong-in, Gyeonggi-Do, Korea).

### 2.2. Preparation of Stock Solution, Calibration Standard (STD), Quality Control (QC) and Internal Standard (ISTD)

Vipadenant stock solution was prepared at a concentration of 1 mg/mL by dissolving 5 mg of vipadenant in 5 mL of dimethyl sulfoxide (DMSO) and a 0.1 mg/mL sub-stock was obtained from 1 mg/mL stock. The working solutions for calibration standards (STDs) and QCs were then prepared by serially diluting the sub-stock using DMSO. 

Seven calibration standards of vipadenant were prepared in duplicate by spiking 20 μL of blank rat plasma with 4 μL of freshly prepared working standard solutions to achieve the final concentrations of 3.02, 9.05, 27.2, 81.5, 244, 733 and 2200 ng/mL, respectively. 

Four levels of QCs were prepared by spiking 20 μL of blank rat plasma with 4 μL of QC solutions freshly prepared from the sub-stock solution of 0.1 mg/mL to obtain a final concentration of low QC (15.0 ng/mL), medium QC (165 ng/mL), high QC (1820 ng/mL) and five-fold diluted QC (5× DQC, 6600 ng/mL). 

A separate verapamil solution (1 mg/mL) was prepared in DMSO and used as an internal standard (ISTD). The ISTD solution containing 20.0 ng/mL of verapamil was freshly prepared in ACN prior to sample preparation.

### 2.3. Sample Preparation—Plasma Samples (Method Qualification Samples and PK Samples)

Twenty microliters of the rat pharmacokinetics samples were placed in cluster tubes. As a make-up solution, 4 μL of DMSO was added to the cluster tube and 100 μL of ACN containing ISTD (verapamil) was also added. The mixture was capped, gently shaken for approximately 1 min and was then centrifuged for 5 min at 12,000 rpm (4 °C). Following the centrifugation, 50 μL supernatant was transferred to a clean test tube and was diluted with 100 μL of distilled water. The resulting mixture was then transferred to an LC-vial and 10 μL was injected to the LC-QTOF-MS. The same procedure was also applied to the STD samples and QC samples. Some PK samples out of the calibration curve (e.g., early time point samples of the intravenous (IV) PK study) were diluted 5-fold with the blank rat plasma during the sample preparation procedure. 

### 2.4. Sample Preparation—In Vitro/Vivo Metabolite Identification

(1) The enzymatic reaction using rat liver microsome (4 mg/mL protein) was initiated by adding cofactors such as NADPH (for oxidative phase I metabolism), UDPGA (for phase II metabolism) and GSH (for phase II glutathione conjugation) at a final concentration of 4, 10 and 0.5 mM, respectively. The cofactor-microsome mixture was pre-incubated at 37 °C for 3 min. 200 μL of the pre-incubated mixture was then transferred to 1.5 mL Eppendorf tube and vipadenant (2.2 μL, 2 mM) or buspirone (2.2 μL, 2 mM, positive control) was added to each tube. The incubation was carried out at 37 °C for 90 min. The reaction was quenched by adding 700 μL of ACN for protein precipitation. The quenched samples were gently shaken and were then centrifuged at 8000 rpm for 10 min and 850 μL of the resulting supernatants was evaporated to dryness under vacuum in a rotary evaporator (Eyela CVE-3110 & UT-1000). Dried residue was re-constituted to 210 μL of DW/MeOH (2:1), shaken, centrifuged at 12,000 rpm for 5 min and the supernatant was transferred to an LC-vial for analysis. 

(2) In vivo metabolite identification samples obtained from the 10 mg/kg oral (PO) PK study and the 2 mg/kg IV PK study were collected according to the Hamilton pooling method [19]. 400 μL of the pooled plasma sample was transferred to a clean tube and 1.3 mL of ACN was added. The pretreated samples were centrifuged at 10,000 rpm for 10 min and 1200 μL of the supernatant was evaporated to dryness under vacuum in a rotary evaporator. The dried residue was re-constituted, shaken and centrifuged in the same manner as the in vitro MetID study. After centrifugation, the supernatant was transferred to an LC-vial for analysis. 

### 2.5. LC-QTOF-MS Condition

The high resolution LC-QTOF-MS system was used for metabolite identification (MetID) as well as quantification of vipadenant. It was consisted of a Shimadzu CBM-20A/LC-20AD chromatographic system (Shimadzu Scientific Instruments, Riverwood Dr, Columbia, SC, USA), with an eksigent CTC HTS PAL auto-sampler (Sciex, Redwood City, CA, USA) and a quadrupole time-of-flight TripleTOF™ 5600 mass spectrometer (Sciex, Redwood City, CA, USA). A Phenomenex^®^ Kinetex XB-C18 column (2.1 × 50 mm for method qualification and 2.1 × 100 mm for MetID) was used as an analytical column for this study. The HPLC mobile phase was consisted of distilled/deionized water containing 0.1% formic acid (phase A) and acetonitrile containing 0.1% formic acid (phase B) with a binary gradient program. The LC-gradient for the quantification and the MetID is summarized in Table 1. The flow rate of the LC system was 0.4 mL/min and the column temperature was set at 55 °C. 

(1) The mass spectrometric conditions for the quantification of vipadenant were optimized as follows; first, product ion scans (TOF-MS/MS) were performed through electro-spray ionization (ESI, positive ion conditions). Then, the product ion with the highest intensity was selected for the quantification under the single reaction monitoring at high sensitivity option (SRMHS). The ion spray voltage (IVSF) was set at 5500 V. The source gas (nebulizer [GS1, ion source gas1] and heater [GS2, ion source gas2]) was set at 50 psi and the source temperature was set at 500 °C with the curtain gas (CUR) flow of 30 L/min. The de-clustering potential (DP) was 70 V and the collision energy (CE) was 15 V. 

(2) The mass spectrometric conditions for the metabolite identification of vipadenant were as follows; first, an information-dependent analysis (IDA) method using high-resolution TOF full scan (ESI, positive ion conditions, *m*/*z* 50 to 1100) was performed. This IDA method included a real time multiple mass defect filtering (MDF) function and a dynamic background subtraction function. Following the TOF full scan, seven unique information-dependent product ion scans (*m*/*z* 50 to 1100) related to the metabolites were performed on the basis of intensity and exclusion parameters. The ion spray voltage (IVSF) was set at 5500 V. The source gas was set at 50 psi and the source temperature was set at 600 °C with the curtain gas (CUR) flow of 30 L/min. The de-clustering potential (DP) was 70 V and the collision energy (CE) was 5V for TOF-MS scan and 20V for the TOF product ion scan. 

### 2.6. Method Qualification

The “fit-for-purpose” method development and qualification were performed. In order to set the range of the calibration curve, a qualification run was conducted over three different days using three different ‘calibration curves in duplicate.’ Calibration curves with seven points were freshly prepared for all data sets. 

Three levels of QCs (low QC [15.0 ng/mL], medium QC [165 ng/mL] and high QC [1820 ng/mL]) were included to evaluate the accuracy and precision of the method. The intra-run accuracy/precision were evaluated using three replicates of QC samples, while the inter-run assay was evaluated using a total of 9 replicates of QC samples.

Dilution integrity evaluation was performed for the PK samples with the concentrations above the upper limit of quantification (ULOQ). A single dilution-QC set consisting of six replicates was evaluated. 

Preliminary stability studies have been conducted to demonstrate that the vipadenant is stable during various storage conditions and plasma pretreatment. Each stability assessment was carried out in blank rat plasma. Three levels of QC (low, medium and high) were used in the short-term, freeze-thaw, long-term and post-preparative stability assessments and two levels of QC (medium & high) were used in stock solution stability assessment. The short term stability assessment was carried out at room temperature (RT) over 4 h and the results obtained were compared with the control (0 min). In the long-term stability assessment, the samples kept frozen at −80 °C for 2 weeks were compared with the freshly prepared control group samples. The freeze-thaw stability was evaluated over three cycles of freezing (−80 °C) and thawing (RT). The post-preparative stability was assessed for the QC samples kept in an auto-sampler (10 °C) for 24 h. As a separate stock-solution stability assessment, the vipadenant stock-solution prepared at 1 mg/mL (DMSO) was stored at −80 °C for 6 months and its stability was evaluated. The stability test results were evaluated by confirming that the mean accuracy (%) of the nominal value which should be within ± 25% range. 

The species-dependent matrix effect was assessed by spiking two different QC samples (medium & high QC in six replicate samples) from four different species (mouse, dog, monkey and human) into each species’ blank plasma. QC samples made in plasma of species other than rat were evaluated against the calibration curve made of rat plasma. The recovery of vipadenant (percentage extraction efficiency) was determined by comparing the mean peak areas of QC samples (fully processed all the way to the final step) with the mean peak areas of fully processed blank samples post-spiked with the solution containing vipadenant at concentrations representing 100% recovery.

### 2.7. Software

Data acquisition and LC-QTOF-MS operation was conducted using Analyst^®^ TF Version 1.6 (Sciex). MultiQuant^®^ Version 2.1.1 (Sciex) was used for the peak integration for vipadenant quantification. PeakView^®^ Version 2.2 and MetabolitePilot™ Version 2.0.2 were used for the structural elucidation of vipadenant metabolites. The descriptive statistics for the qualification studies were calculated with Excel 2015 (Microsoft). Pharmacokinetic parameters were calculated in a non-compartmental analysis using WinNonlin^®^ version 8.0.0 (Certara, Princeton, NJ, USA).

### 2.8. Application for Animal Study

Male sprague-dawley rats (300 ± 10 g) were purchased from the Samtako Biokorea Co. (Gyeonggi, Korea) and housed in groups of 3~4 per cage and given standard rodent chow. All rats were kept for at least one week prior to starting the PK study and fasted 12 h prior to drug administration. Rats were distributed into four different groups (three rats per group; 1 & 2 mg/kg IV group and 2 & 5 mg/kg PO group). Blood was drawn into the heparinized tubes after IV (1 and 2 mg/kg) or PO (2 and 5 mg/kg) administration and was immediately centrifuged at 10,000 rpm for 5 min. The sampling time points were 0, 2, 5, 15, 30, 60, 90, 120, 240, 360 and 480 min for IV administration and 0, 5, 15, 30, 60, 90, 120, 240, 360 and 480 min for PO administration. The supernatant of the centrifuged samples (plasma) were transferred to the clean tubes and stored at −20 °C until analysis. 

A separate in vivo study was conducted for in vivo MetID purpose. One set of rats (*n* = 3) was administered orally (10 mg/kg). After administration, blood sampling was performed in the same manner as the previous PK studies. The collected plasma samples were stored at −20 °C until analysis. 

All experiment performed on the rats were approved by abiding the animal care protocol (no. CNU-01104) from Chungnam National University. The procedures were abided by the guidelines established by the Association for Assessment and Accreditation of Laboratory Animal Care International (AAALAC International).

## 3. Results

### 3.1. Method Development

#### 3.1.1. Sample Preparation

In the early preclinical stage, it is important to develop a sensitive assay with a simple and good selectivity for pharmacokinetics as well as drug metabolism research [20,21,22,23]. The most frequently used pretreatment methods at the early drug discovery stage include protein precipitation (PPT), liquid-liquid extraction (LLE) and solid-phase extraction (SPE) [21,22,23]. In this study, we aimed for the development of a simple and sensitive sample preparation procedure with the sample volumes as low as possible (e.g., 20 μL). Although the protein precipitation method has disadvantages associated with the extraction efficiency during the pretreatment process [24,25], there was no problem with the recovery in our experiments using vipadenant (recovery: 97.7~102.1%). 

#### 3.1.2. Optimization of the LC-MS System

The optimum LC-QTOF-MS condition for vipadenant was evaluated in the positive ion mode using various combinations of mobile phase conditions and HPLC columns. The best condition was observed with DW/ACN (each containing 0.1% formic acid) as mobile phase and a Phenomenex^®^ Kinetex XB-C18 column (2.1 × 50 mm, 2.6 μm). 

The major ion of vipadenant during TOF full scan was the protonated [M + H]^+^ ion at *m*/*z* 322.1. Also, two major product ions were observed by the TOF product ion scan and the most abundant product ion (*m*/*z* 120.1) was selected for the quantification using the single reaction monitoring at high sensitivity option (SRMHS) transition for vipadenant (322.1 → 120.1 & 203.1). 

The information-dependent analysis (IDA) method was also optimized for all metabolites derived from in vitro/vivo MetID studies. The same mobile phase condition was used for the method development, while a longer column (Phenomenex^®^ Kinetex XB-C18 column, 2.1 × 100 mm, 2.6 μm) with a different LC-gradient (Table 1) was used for the MetID sample analysis.

### 3.2. Method Qualification

#### 3.2.1. Calibration Curve, Accuracy, Precision

**Figure 2:**
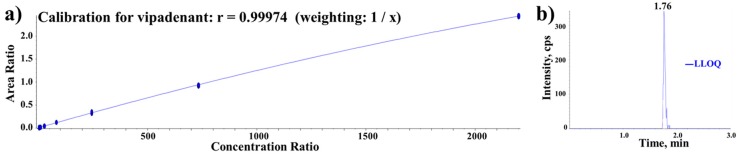
(**a**) Calibration curve of vipadenant and (**b**) Lower limit of quantification (LLOQ) in rat plasma.

The lower limit of quantification (LLOQ) of the assay was determined to be 3.02 ng/mL. The calibration curve was evaluated in the range of 3.02~2200 ng/mL and the quadratic regression (weighted 1/concentration) was used for the best fit of the curve with a correlation coefficient ≥0.997. The calibration curve and the chromatogram of the LLOQ level spiked in plasma are shown in Figure 2.

Three levels of QC samples were used for the determination of assay performance by assessing the precision (RSD (% CV)) and the mean accuracy (%). The results are shown in Table 2. The back calculated concentrations for all the samples met the acceptance criteria within ± 25% of the nominal value, ranging from 94.9 to 123.3% for intra-run assay and 101.3 to 108.1% for inter-run assay with precision values (RSD (% CV)) within 25%, which is acceptable for early stage pre-clinical study. 

#### 3.2.2. Preliminary Stability

The preliminary stability test showed that vipadenant in rat plasma QC sample was stable for at least 4 h at room temperature, which is sufficient enough for the sample preparation process. Vipadenant was stable in three cycles of freeze-thawing (RT vs. −80 °C) in rat plasma and was also stable in a long-term stability test (−80 °C storage condition) for at least 2 weeks. The processed samples were stable for at least 24 h in an auto-sampler storage condition (10 °C) and the stock solution prepared in DMSO showed stability for 6 months. Each stability results are shown in Table 3. 

#### 3.2.3. Species-Dependent Matrix Effect

At the pre-clinical testing phase, various studies can be done in both rodent and non-rodent animal models. If there was little or no species-dependent matrix effect, the calibration curve from the rat plasma could be applicable to studies of other pre-clinical species PK samples. Table 4 shows no significant species-dependent matrix effect from other species when compared to rat plasma samples. 

### 3.3. Application

#### 3.3.1. Pharmacokinetic Study

**Figure 3:**
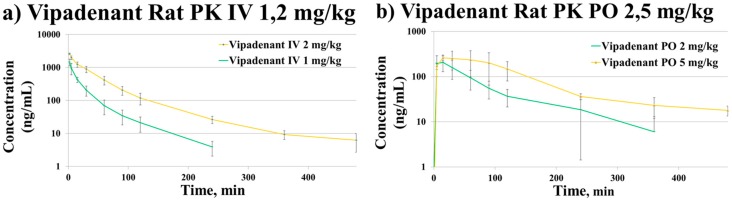
Time-concentration profiles of vipadenant from (**a**) IV (intravenous) 1, 2 mg/kg study and (**b**) PO (oral) 2, 5 mg/kg study.

The developed LC-QTOF-MS method was successfully applied to determine the PK parameters after PO (2, 5 mg/kg) and IV (1, 2 mg/kg) administration of vipadenant in rats. During the sample preparation of the PK samples, QC samples were included for the assurance of the analytical run. All the PK samples from PO and IV studies were within the range of the qualified calibration range (3.02–2200 ng/mL). However, the peak concentrations from early time-point study samples (2 min) of the IV 2 mg/kg study were out of the calibration range and therefore 5-fold dilution was used using blank rat plasma. The time-concentration profile of vipadenant is shown in Figure 3. The PK parameters from each of the studies were calculated with the non-compartmental analysis using WinNonlin (version 8.0.0) and the results are summarized in Table 5. 

The PK results showed that vipadenant has a low to moderate clearance in the rat IV study. The average bioavailability of vipadenant was 30.4 ± 8.9%. In the IV PK studies, the area-under-the curve (AUC) value was not increased dose-proportionally and the clearance of vipadenant was decreased almost two fold (37.8 mL/min/kg → 22.5 mL/min/kg) when the IV dose was increased from 1 mg/kg to 2 mg/kg. Based on this observation, vipadenant appears to exhibit non-linear PK even in these low dose levels. 

#### 3.3.2. In Vitro/Vivo Metabolite Identification

**Figure 4:**
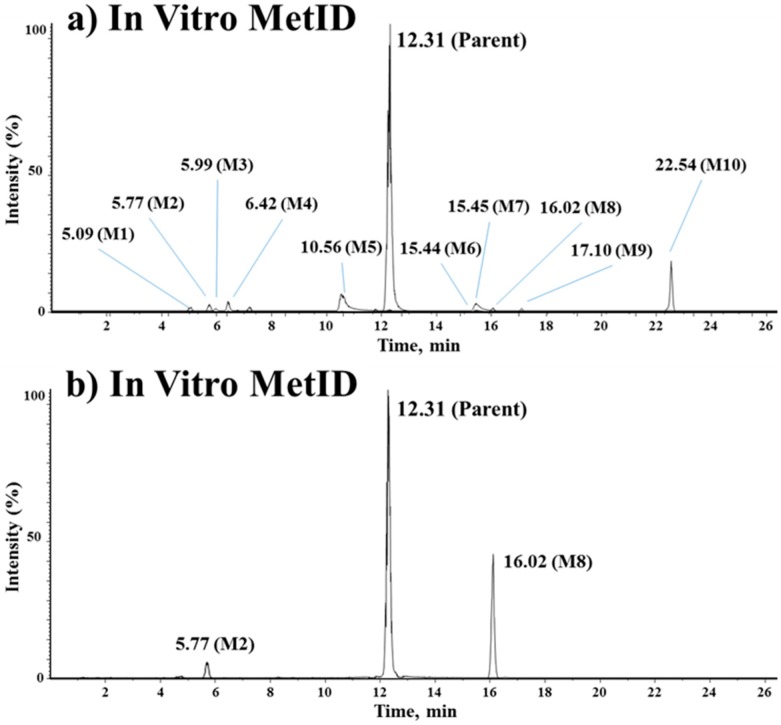
Extracted ion chromatogram of representative MetID samples. (**a**) in vitro (rat liver microsome) and (**b**) in vivo MetID (rat pharmacokinetic study) of vipadenant.

**Figure 5:**
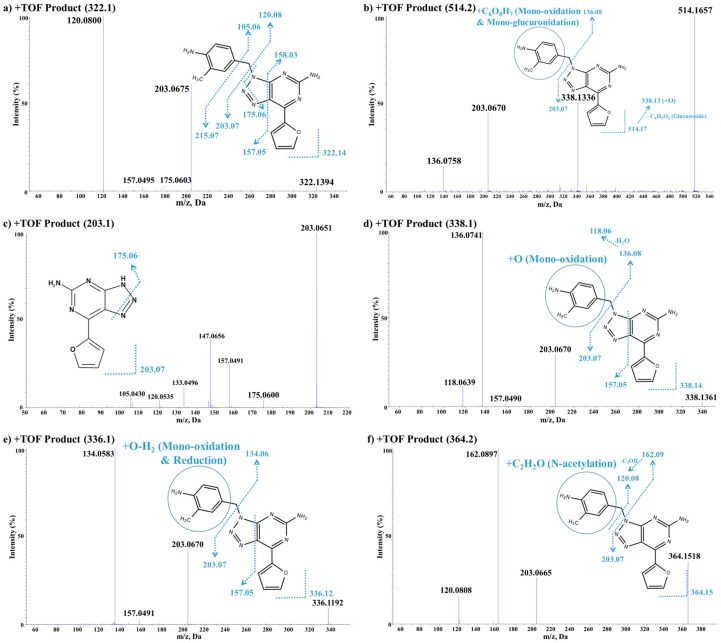
Identification of in vitro/vivo metabolites based on vipadenant. (**a**) TOF-MS/MS scan of vipadenant (*m*/*z* 322.1); (**b**) TOF-MS/MS scan of ‘mono-oxidation and mono-glucuronidation’ metabolite (M1: *m*/*z* 514.1); (**c**) TOF-MS/MS scan of the metabolite with a ‘loss of C_8_H_9_N’ moiety from vipadenant (M2: *m*/*z* 203.1), (**d**) TOF-MS/MS scan of ‘mono-oxidation’ metabolites (M3, M4, M5, M7: *m*/*z* 338.1); (**e**) TOF-MS/MS scan of metabolites with ‘mono-oxidation followed by reduction’ (M6, M9, M10: *m*/*z* 336.1) and (**f**) TOF-MS/MS scan of ‘acetylated’ metabolites (M8: *m*/*z* 364.1).

**Figure 6:**
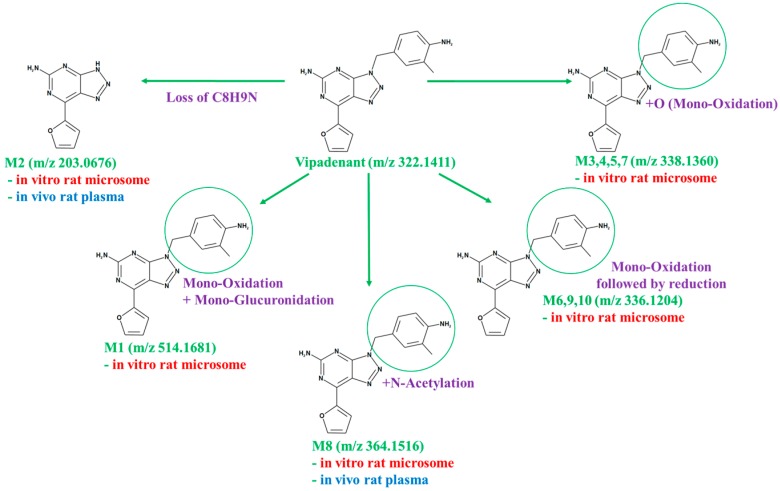
Metabolic pathways of vipadenant in vitro/vivo.

The metabolites of vipadenant were investigated using an LC-QTOF-MS assay. Under the current experimental conditions, ten metabolite peaks were observed from the in vitro study, while two peaks were observed from the in vivo study. The TOF-MS chromatographic separation of vipadenant and its metabolites are shown in Figure 4. Each of the metabolites were analyzed using product ion scans (TOF-MS/MS) in positive ion mode to elucidate the structures (Figure 5.) and the metabolic pathways of vipadenant is described in Figure 6. 

##### Vipadenant

Vipadenant showed a molecular ion [M + H]^+^ at *m*/*z* 322. The TOF-MS/MS analysis of *m*/*z* 322 leads to the formation of fragment ions at *m*/*z* 203, 175, 157 and 120. The fragment ion *m*/*z* 203 is formed by the neutral loss of C_8_H_9_N from *m*/*z* 322. The subsequent loss of N_2_ from *m*/*z* 203 leads to the formation of *m*/*z* 175. The detailed fragmentation information and TOF-MS/MS data of vipadenant are shown in Figure 5a.

##### Metabolite M1

M1 showed a molecular ion [M + H]^+^ at *m*/*z* 514 which was 192 (16 + 172) amu higher than the molecular ion of vipadenant. The TOF-MS/MS analysis of *m*/*z* 514 leads to the formation of fragment ions at *m*/*z* 338, 203 and 136. The unchanged fragment ion *m*/*z* 203 suggest that metabolism has occurred in 2,4-dimethylaniline moiety of vipadenant. The fragment ion 338 represents the mono-oxidation moiety of vipadenant, which is formed by the loss of glucuronide (*m*/*z* 176) from *m*/*z* 514. The subsequent neutral loss of C_8_H_9_N from *m*/*z* 338 leads to the formation of *m*/*z* 136. The accurate mass measured for M1 and the difference between theoretical values are summarized in Table 6. These results suggest that M1 was a mono-oxidation & mono-glucuronidation metabolite of vipadenant. The detailed fragmentation information and TOF-MS/MS data of M1 are shown in Figure 5b.

##### Metabolite M2

M2 showed a molecular ion [M + H]^+^ at *m*/*z* 203 which was 120 amu lower than the molecular ion of vipadenant. The TOF-MS/MS analysis of *m*/*z* 203 leads to the formation of fragment ions at *m*/*z* 175, 157, 147, 133, 120 and 105. The loss of N_2_ leads to the formation of *m*/*z* 175. By comparing its fragments with those of vipadenant as well as its high resolution MS data, M2 was confirmed to be 7-(2-Furyl)-3H-[1,2,3]triazolo[4,5-d]pyrimidin-5-amine and it was made by the neutral loss of C_8_H_9_N from vipadenant. The accurate mass measured for M2 and the difference between the theoretical values are summarized in Table 6, while the detailed fragmentation information and TOF-MS/MS data are shown in Figure 5c.

##### Metabolites M3, M4, M5 and M7

These metabolites (Figure 5d) showed a molecular ion [M + H]^+^ at *m*/*z* 338 which was 16 amu higher than the molecular ion of vipadenant, suggesting a mono-oxidation is taking place on vipadenant. The TOF-MS/MS analysis of *m*/*z* 338 leads to the formation of fragment ions at *m*/*z* 203, 157, 136 and 118. The unchanged fragment ions *m*/*z* 203 and 157 suggest that metabolism has occurred in 2,4-dimethylaniline moiety of vipadenant. The fragment ion at *m*/*z* 136 is also 16 amu higher than *m*/*z* 120 found in vipadenant suggesting that mono-oxidation was occurred in 2,4-dimethylaniline moiety of vipadenant. All three metabolites had differences in the retention time (M3 = 5.99, M4 = 6.42, M5 = 10.6, M7 = 15.5 min), meaning that the metabolism was occurred in different sites in the 2,4-dimethylaniline moiety. The accurate mass measured for these metabolites and the difference between the theoretical values are summarized in Table 6.

##### Metabolites M6, M9 and M10

These metabolites (Figure 5e) showed a molecular ion [M + H]^+^ at *m*/*z* 336 which was 14 amu higher than the molecular ion of vipadenant, suggesting a mono-oxidation followed by reduction is taking place on vipadenant. The TOF-MS/MS analysis of *m*/*z* 336 leads to the formation of fragment ions at *m*/*z* 203, 157 and 134. The unchanged fragment ions *m*/*z* 203 and 157 suggest that metabolism has occurred in 2,4-dimethylaniline moiety of vipadenant. The fragment ion *m*/*z* 134 is also 14 amu higher than *m*/*z* 120 found in vipadenant suggesting that mono-oxidation followed by reduction was occurred. All three metabolites had a difference in the retention time (M6 = 15.4, M9 = 17.1, M10 = 22.5 min), meaning that the metabolism is occurred in different sites in the 2,4-dimethylaniline moiety. The accurate mass measured for these metabolites and the difference between the theoretical values are summarized in Table 6.

##### Metabolite M8

M8 showed a molecular ion [M + H]^+^ at *m*/*z* 364 which was 42 amu higher than the molecular ion of vipadenant. The TOF-MS/MS analysis of *m*/*z* 364 leads to the formation of fragment ions at *m*/*z* 203, 162 and 120. The unchanged fragment ion *m*/*z* 203 suggest that metabolism has occurred in 2,4-dimethylaniline moiety of vipadenant. The fragment ion *m*/*z* 162 is 42 amu higher than *m*/*z* 120 found in vipadenant. These results suggest that M8 was an acetylation (+C_2_H_2_O) metabolite of vipadenant. The accurate mass measured for M8 and the difference between the theoretical values are summarized in Table 6, while the detailed fragmentation information and TOF-MS/MS data are shown in Figure 5f.

## 4. Discussion & Conclusions

Vipadenant is a highly potent non-xanthine-based pyrimidine-amine type of A2a receptor antagonist and is being developed as a potential immune checkpoint inhibitor. To our best knowledge, there has been no report on the development of quantitative methods for vipadenant in plasma samples as well as metabolite identification/profiling so far.

Since there have been no studies on the quantitative analysis of vipadenant PK samples so far, we developed a simple and reproducible LC-QTOF-MS assay with high sensitivity for vipadenant. The lower limit of quantification (LLOQ) was 3.02 ng/mL and the calibration linearity was demonstrated at the range of 3.02–2200 ng/mL. Assay qualification was performed in accordance to the accuracy and precision values of intra/inter run. A preliminary stability test including short-term (4 h), freeze-thaw (3 cycles), long-term stability (2 weeks), stock solution storage (6 months) and post-preparative stability (24 h, auto-sampler storage) shows the precision and accuracy values within the acceptance criteria of ± 25%, which are sufficient enough for the early stage drug development and preclinical study [26,27].

The assay was successfully applied for the rat PK study after intravenous and oral administration of vipadenant. Unlike other A2a receptor inhibitors (e.g., SCH58261 or ZM241385), the bioavailability of vipadenant was relatively acceptable to be administered orally [17,18]. The clearance of vipadenant in the IV study was low to moderate which is similar to the in-house metabolic stability study (in vitro intrinsic clearance = 20.0 mL/min/kg [rat liver microsome]). However, non-linear PK properties were observed at these low dose levels. We hypothesize that one of the clearance pathways would be likely saturated even in the dose range of IV 1~2 mg/kg and PO 2~5 mg/kg and the similar phenomenon was also observed in a human trial for the receptor occupancy study [18].

The metabolites of vipadenant were identified by the LC-QTOF-MS analysis using IDA method. The MetID study showed difference in the formation of metabolites in vitro and in vivo. In our experimental condition, 5 types of metabolites were identified from the in vitro study with rat liver microsome (‘mono-oxidation,’ ‘mono-oxidation followed by reduction,’ ‘mono-oxidation + mono-glucuronidation,’ ‘*N*-acetylation’ and ‘the loss of C_8_H_9_N’) and the major portions were the phase 1 metabolites such as ‘mono-oxidation’ and ‘mono-oxidation followed by reduction.’ In case of in vivo MetID study, only two types of metabolites (‘*N*-acetylation’ and ‘the loss of C_8_H_9_N’) were observed from the in vivo PK samples collected after PO administration at a dose of 10 mg/kg. The major metabolites identified in the in vivo samples were the acetyl-conjugates, the phase 2 metabolites of the benzyl-amine moiety of the vipadenant. The reason for the difference of in vitro/vivo metabolites would be likely due to the limitation of metabolic enzymes or cofactors existing in the in vitro liver microsome compared to the whole in vivo system [28,29,30,31,32].

In conclusion, we developed a sensitive, simple and reproducible LC-QTOF-MS method to quantify vipadenant in PK samples and also evaluated the in vitro/vivo metabolites of vipadenant for the first time. This assay could be applied for the future development of vipadenant or its analogs that have similar structural moieties as potential immune checkpoint inhibitors.

## Figures and Tables

**Table 1:** The mobile phase conditions for LC (Liquid-Chromataography) gradient.

**1. LC Gradient for Quantification**	
**Time (min)**	**Mobile Phase B (%)**
0	10
0.5	10
0.9	95
1.5	95
1.6	10
3.0	10
**2. LC Gradient for MetID**	
**Time (min)**	**Mobile Phase B (%)**
0	5
1.5	5
18	25
23	50
23.5	95
27	95
27.5	5
32	5

**Table 2:** Quality control results and statistics from the intra/inter-run assays for vipadenant.

**Intra-Run Assay**					
**Run**	**Nominal QC Concentration (ng/mL)**	**Calculated Concentration (ng/mL)**	**Mean Accuracy (%)**	**Precision (% CV)**	***n***
Day 1	15	16.9	112.8	12.1	3
	165	175.2	106.2	7.9	
	1820	1793.4	98.5	3.2	
Day 2	15	17.3	114.8	9.6	3
	165	164.2	99.2	6.3	
	1820	1727.6	94.9	5.3	
Day 3	15	18.5	123.3	4.9	3
	165	187.1	113.4	6.9	
	1820	1923.1	105.7	5.3	
**Inter-Run Assay (Day 1~3)**					
**Nominal QC Concentration (ng/mL)**		**Calculated Concentration (ng/mL)**	**Mean Accuracy (%)**	**Precision (% CV)**	***n***
15		16.2	108.1	11.3	9
165		178.1	107.8	7.4	
1820		1843.7	101.3	4.6	

**Table 3:** Preliminary stability results for vipadenant.

Freeze-Thaw, Long-Term and Post-Preparative Stability Assessment				
Stability Test	Nominal QC Concentration (ng/mL)	Calculated Concentration (ng/mL)	Mean Accuracy (%)	Precision (% CV)
Short term (4 h, RT, *n* = 3)	15	14.7	97.8	11.6
	165	149.4	90.5	10.9
	1820	1685.6	92.6	3.9
Freeze-thaw (3 cycles, −80 °C, *n* = 3)	15	17.8	118.8	5.2
	165	145.8	88.5	0.4
	1820	1671.6	91.1	1.7
Long-term (2 weeks, −80 °C, *n* = 3)	15	16.8	111.8	3.3
	165	171.8	104.1	2.7
	1820	1887.7	103.7	4.8
Post-preparative (24 h, 4 °C, *n* = 6)	15	17.3	114.8	9.6
	165	164.2	99.2	6.3
	1820	1727.6	94.9	5.3
Stock storage (6 months, −80°C, *n* = 3)	165	169.1	102.5	3.3
	1820	1739.2	95.6	6.1

**Table 4:** The Species-dependent matrix effect of vipadenant.

Species-Dependent Matrix Effect Assessment (5 Species)						
Species	QC Medium (165 ng/mL, *n* = 3)			QC High (1820 ng/mL, *n* = 3)		
	Mean Concentration (ng/mL)	Mean Accuracy (%)	Precision (% CV)	Mean Concentration (ng/mL)	Mean Accuracy (%)	Precision (% CV)
Control (Rat)	153.2	92.7	2.9	1788.0	98.3	5.9
Mouse	159.7	96.6	6.1	1881.6	103.5	9.6
Dog	159.8	96.7	4.8	1879.4	103.4	7.9
Monkey	163.7	99.1	3.3	1740.3	95.7	3.1
Human	174.0	105.3	6.6	1857.9	102.2	3.4

**Table 5:** Pharmacokinetic parameters of vipadenant from IV/PO PK study.

PK Parameters of Vipadenant								
PK Study	Dose (mg/kg)	*T*_1/2_ (min)	*T*_max_ (min)	*C*_0_ or *C*_max_ (ng/mL)	AUC_last_ (min ng/mL)	CL (mL/min/kg)	Vss (mL/kg)	BA (%)
PO	2	65.2 ± 20.2	7.5 ± 5.0	229.7 ± 88.0	16716.4 ± 7245.0	-	-	30.4 ± 8.9
	5	115.6 ± 59.0	30.0 ± 26.0	296.0 ± 87.3	53196.4 ± 4067.6	-	-	
IV	1	48.0 ± 5.8	2.6 ± 1.3	2213.7 ± 1155.3	27060.9 ± 5826.5	37.8 ± 7.2	1082.9 ± 222.5	
	2	71.2 ± 8.5	2.0 ± 0.0	3091.0 ± 221.6	90694.2 ± 18814.6	22.5 ± 4.3	1209.3 ± 174.4	

**Table 6:** Characteristics of the in vitro/vivo vipadenant MetID results by LC-QTOF-MS/MS assay.

**In Vitro MetID Result of Vipadenant**						
**Peak ID**	**Name**	**Formula**	**R.T (min)**	***m*/*z***	**Nominal Mass Change (Da)**	**Error ppm**
Parent	Vipadenant [M + H]^+^	C_16_H_15_N_7_O	12.3	322.1410	-	−0.2
M1	Mono-oxidation + mono-glucuronidation [M + H]^+^	C_22_H_23_N_7_O_8_	5.1	514.1656	+192	−4.8
M2	Loss of C_8_H_9_N [M + H]^+^	C_8_H_6_N_6_O	5.8	203.0651	−120	−0.9
M3	Mono-oxidation [M + H]^+^	C_16_H_15_N_7_O_2_	6.0	338.1363	+16	0.9
M4	Mono-oxidation [M + H]^+^	C_16_H_15_N_7_O_2_	6.4	338.1338	+16	−6.5
M5	Mono-oxidation [M + H]^+^	C_16_H_15_N_7_O_2_	10.6	338.1362	+16	0.6
M6	Mono-oxidation followed by reduction [M + H]^+^	C_16_H_13_N_7_O_2_	15.4	336.1193	+14	−3.1
M7	Mono-oxidation [M + H]^+^	C_16_H_15_N_7_O_2_	15.5	338.1338	+16	5.3
M8	*N*-Acetylation [M + H]^+^	C_18_H_17_N_7_O	16.0	364.1527	+42	2.9
M9	Mono-oxidation followed by reduction [M + H]^+^	C_16_H_13_N_7_O_2_	17.1	336.1192	+14	−3.4
M10	Mono-oxidation followed by reduction [M + H]^+^	C_16_H_13_N_7_O_2_	22.5	336.1187	+14	−4.9
**In Vivo MetID Result of Vipadenant**						
**Peak ID**	**Name**	**Formula**	**R.T (min)**	***m*/*z***	**Nominal Mass Change (Da)**	**Error ppm**
Parent	Vipadenant [M + H]^+^	C_16_H_15_N_7_O	12.3	322.1417	-	1.9
M2	Loss of C_8_H_9_N [M + H]^+^	C_8_H_6_N_6_O	5.8	203.0673	−120	−1.4
M8	*N*-Acetylation [M + H]^+^	C_18_H_17_N_7_O	16.0	364.1526	+42	2.6
